# Common metabolite for microangiopathy in Japanese and European populations

**DOI:** 10.1111/jdi.14189

**Published:** 2024-03-22

**Authors:** Nobuhiro Shojima, Toshimasa Yamauchi

**Affiliations:** ^1^ Department of Diabetes and Metabolic Diseases, Graduate School of Medicine The University of Tokyo Tokyo Japan

Blood metabolomics profiling has emerged as a powerful approach for investigating metabolic diseases^1^. Blood is readily accessible and contains metabolites derived from various physiological systems. Mass spectrometry and nuclear magnetic resonance (NMR) spectroscopy offers an analytical platform for the comprehensive analysis of blood metabolites, including proteins, lipids, peptides, and glycans. Metabolites represent the end product of biological processes and are sensitive to environmental exposures. Thus, it is expected that the levels of metabolites could indicate a disease risk at an early stage. It is important to replicate and validate findings from untargeted metabolomics studies.

Tomofuji *et al*.^2^ revealed the serum metabolite signatures of persons with type 2 diabetes mellitus with both diabetic retinopathy (DR) and diabetic kidney diseases (DKD) using a comprehensive untargeted metabolomics approach combining capillary electrophoresis time‐of‐flight mass spectrometry (CU‐TOFMS) and liquid chromatography TOFMS^2^. They compared the abundance of 364 metabolites between persons with type 2 diabetes mellitus with both DR and DKD (*N* = 141) and those without either DR or DKD (*N* = 159). They revealed that five metabolites including N‐acetylneuraminic acid were associated with complications of type 2 diabetes in a Japanese population (cyclohexylamine, *P* = 4.5 × 10^−6^; 1,2‐distearoyl‐glycero‐3‐phosphocholine, *P =* 7.3 × 10^−6^; piperidine, *P* = 4.8 × 10^−4^; N‐acetylneuraminic acid, *P* = 5.1 × 10^−4^; stearoyl ethanolamide, *P =* 6.8 × 10^−4^).

Ancel *et al*.^3^ reported the serum metabolite signatures of persons with type 2 diabetes mellitus with both diabetic retinopathy and diabetic kidney diseases with a comprehensive nontargeted metabolomics and lipidomics approach^3^. They compared the abundance of 563 metabolites between persons with type 2 diabetes mellitus with both DR and DKD (*N* = 53) and those without either DR or DKD (*N* = 61). They revealed that four metabolites including N‐acetylneuraminate were associated with complications of type 2 diabetes mostly in European populations (methylguanidine, *P* < 0.001, 1.89 fold change; N‐acetylneuraminate, *P* < 0.001, 1.73 fold change; arabinose, *P* < 0.001, 2.37 fold change; mevalolactone *P* < 0.001, 1.30 fold change), which is consistent with the results of Tomofuji *et al*. (Figure 1).

**Figure 1:**
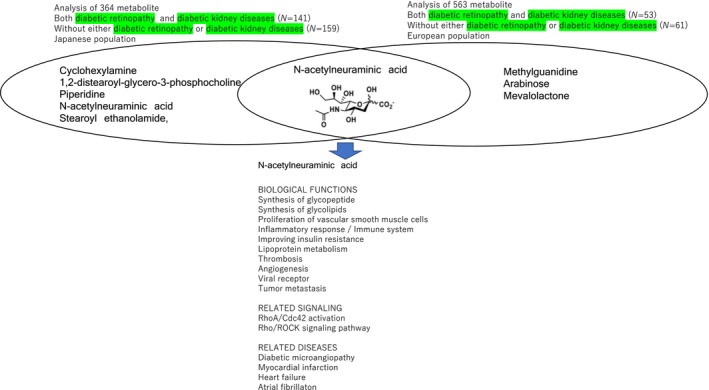
Untargeted metabolomics across populations discovered N‐acetylneuraminic acid to be a potential biomarker and/or a therapeutic target for diabetic retinopathy and diabetic kidney diseases.

N‐acetylneuraminic acid, also known as sialic acid, is one of the commonly distributed natural carbohydrates, and it is also a basic component of many glycopeptides, with a role in monocyte/macrophage migration through binding to the Rho A and Cdc42 and activating the Rho/Rho‐associated coiled‐coil containing protein kinase (ROCK) signaling pathway^4^. N‐acetylneuraminic acid belongs to the monosaccharide family with a 6‐carbon main chain and a high degree of structural diversity, as a viral receptor. It is closely related to malignant transformation and cancer metastasis and invasion. The active metabolites of oseltamivir play a role by inhibiting neuraminidase 1. The contribution of N‐acetylneuraminic acid to the pathogenesis of macro and microvascular complications via biological processes includes inflammatory reaction, disrupting iron metabolism, and promoting platelet thrombosis.

Serum N‐acetylneuraminic acid is reported to be associated with myocardial injury, and prognosis in acute coronary syndrome using data from a total of 766 patients (537 with unstable angina, 100 with myocardial infarction, 129 without coronary artery disease)^5^. The receiver operating characteristic curve analysis showed that a higher serum N‐acetylneuraminic acid was potentially associated with myocardial infarction and high‐risk stratification in acute coronary syndrome patients. Logistic analysis identified only elevated serum N‐acetylneuraminic acid as an independent predictor of major adverse cardiac events (MACEs) in these patients (odds ratio: 1.003, confidence interval: 1.002–1.005, *P* < 0.001). These findings suggest that the serum level of N‐acetylneuraminic acid is related to the clinical prognosis of acute coronary syndrome. Given that there were associations between cardiovascular complications and retinal and renal complications, N‐acetylneuraminic acid complications which could contribute to the shared etiology among the cardiovascular retinal and renal complications of persons with type 2 diabetes mellitus.

The results of metabolomic studies demonstrated that serum N‐acetylneuraminic acid is associated with diabetic microvascular complications in persons with type 2 diabetes mellitus in Japanese and European populations. It may reflect the severity of micro and macrovascular complications and predict a poor prognosis. Thus, N‐acetylneuraminic acid may become a biomarker for the diagnosis and risk stratification of persons with type 2 diabetes mellitus, and targeted N‐acetylneuraminic acid inhibition may represent a novel therapeutic strategy for type 2 diabetes mellitus complications. Longitudinal metabolomic studies about diabetic microvascular complication is needed to establish the correlation and predictive value of N‐acetylneuraminic acid. Whether pharmacological interventions to inhibit N‐acetylneuraminic acid can improve the prognosis of diabetic complications will need to be determined in future clinical trials. Studies are needed to further our understanding of the mechanisms and therapeutic potential of treatments targeting N‐acetylneuraminic acid in persons with type 2 diabetes mellitus with vascular complications.

## DISCLOSURE

Toshimasa Yamauchi is an Editorial Board member of *Journal of Diabetes Investigation* and a co‐author of this article. To minimize bias, he is excluded from all editorial decision‐making related to the acceptance of this article for publication.

The authors declare no financial support or relationships that may pose a conflict of interest.

Approval of the research protocol: N/A.

Informed consent: N/A.

Registry and the registration no. of the study/trial: N/A.

Animal studies: N/A

## References

[1] Fuller H , Zhu Y , Nicholas J , *et al*. Metabolomic epidemiology offers insights into disease aetiology. Nat Metab 2023; 5: 1656–1672. 37872285 10.1038/s42255-023-00903-x PMC11164316

[2] Tomofuji Y , Suzuki K , Kishikawa T , *et al*. Identification of serum metabolome signatures associated with retinal and renal complications of type 2 diabetes. Commun Med 2023; 3: 5. 36624208 10.1038/s43856-022-00231-3 PMC9829655

[3] Ancel P , Martin JC , Doukbi E , *et al*. Untargeted multiomics approach coupling lipidomics and metabolomics profiling reveals new insights in diabetic retinopathy. Int J Mol Sci 2023; 24: 12053. 37569425 10.3390/ijms241512053 PMC10418671

[4] Kooner AS , Yu H , Chen S , *et al*. Synthesis of N‐glycolylneuraminic acid (Neu5Gc) and its glycosides. Front Immunol 2019; 100: 2004. 10.3389/fimmu.2019.02004 PMC6724515 31555264

[5] Li MN , Qian SH , Yao ZY , *et al*. Correlation of serum N‐acetylneuraminic acid with the risk and prognosis of acute coronary syndrome: A prospective cohort study. BMC Cardiovasc Disord 2020; 20: 404. 32912159 10.1186/s12872-020-01690-z PMC7488474

